# Levels of Circulating TIMP-2 and MMP2-TIMP2 Complex Are Decreased in Squamous Cervical Carcinoma

**DOI:** 10.1155/2010/179351

**Published:** 2010-06-29

**Authors:** Talvensaari-Mattila Anne, Turpeenniemi-Hujanen Taina

**Affiliations:** ^1^Department of Obstetrics and Gynecology, Oulu University Hospital, University of Oulu, P.O. Box 24, 90029 OYS Oulu, Finland; ^2^Department of Oncology and Radiotherapy, Oulu University Hospital, University of Oulu, P.O. Box 24, 90029 OYS Oulu, Finland

## Abstract

*Background*. The role of matrix metalloproteinase-2 and -9 (MMP-2, MMP-9) in matrix degradation and metastasis has been described in various tumors. Their action is inhibited by their natural tissue inhibitor molecules TIMP-1 and -2. *Methods*. The study population consisted of 12 squamous cervical carcinoma patients and 27 healthy volunteer control patients. MMP-9, MMP-2-TIMP-2 complex, TIMP-1, and TIMP-2 were analyzed from serum samples using enzyme-linked immunoassay (ELISA). *Results*. The mean levels of serum TIMP-2 and of MMP-2-TIMP-2 complex were higher in healthy controls compared to patients with a malignant tumor. Serum TIMP-2 values decreased significantly from healthy controls (median 323 *μ*g/l, range 305–342 *μ*g/l) to malignant (median 136 *μ*g/l, range 120–151 *μ*g/l) squamous cervical carcinoma patients (*P* < .000). Also, serum proMMP2-TIMP2 complex values decreased from control patients to squamous cervical carcinoma patients (*P* < .006). *Conclusion*. This paper shows that the levels of circulating TIMP-2 and that of MMP-2-TIMP-2 complex are lower in squamous cervical carcinoma patients than in healthy women.

## 1. Introduction

Cervical cancer is the second most common malignant disease among women worldwide [1]. However, its incidence and mortality rates have been decreasing in many highly developed countries, for example, the Nordic countries, mainly due to organized Pap smear screening [2].

Members of the matrix metalloproteinase (MMP) family have the ability to degrade macromolecules of the extracellular matrix, and they are responsible for tumor invasion and infiltration. They are a family of zinc and calcium-dependent proteinases, and they are secreted in the proenzyme form [3]. Besides their role in tumor invasion and metastasis, MMPs are involved in various physiological connective tissue remodeling processes, such as ovulation and wound healing [4].

Matrix metalloproteinases form an enzyme family that takes part in virtually all events of extracellular matrix remodeling and turnover. Cancer cells capable of producing these enzymes, especially of gelatinases MMP-2 and -9, have been shown to have increased invasion potential in several malignancies [5, 6]. Their action is inhibited by their natural tissue inhibitor molecules TIMP-1 and -2.

There are no previous studies on circulating metalloproteinases and squamous cervical carcinoma. The aim of this paper was to evaluate the levels of circulating metalloproteinases and their inhibitors in squamous cervical carcinoma patients and healthy women.

## 2. Materials and Methods

The patient material consisted of 12 early-stage (FIGO stage IB and IIA) squamous cervical carcinoma patients who underwent surgery at Oulu University Hospital during the years from 1992 to 1995 and 27 healthy volunteer control patients. The serum samples were collected by taking venous blood samples from the patients at the time of the primary squamous cervical cancer diagnosis prior to surgery. The samples were collected in glass tubes that did not contain any artificial coagulation activators. They were allowed to coagulate, centrifuged at 3000 rpm for 10 minutes, and the native serum obtained was separated and frozen at −20° until used. 

All patients with cervical carcinoma were treated with preoperative intracavitary brachytherapy, followed by radical hysterectomy and lymph node dissection. The majority of the patients received postoperative radiation. Additional platinol-based chemotherapy was given if pelvic lymph node metastases were found in the operation. The patients' median age was 52 years, ranging from 31 to 80, at the moment of diagnosis. The follow-up time for each patient was 120 months at the minimum. 

### 2.1. Assay for the Immunoreactive Proteins of MMP-2-TIMP-2 Complex, MMP-9, TIMP-1, and TIMP-2

 The immunoreactive proteins for MMP-2-TIMP-2 -complex, MMP-9, TIMP-1, and TIMP-2 were assayed from the sera of the patients with squamous cervical carcinoma and from that of healthy volunteers using enzyme-linked immunoassay (ELISA). The quantification of the total protein of MMP-2-TIMP-2 complex, MMP-9, TIMP-1, or TIMP-2 was performed using standard protocols [7]. Briefly, the ELISAs were performed on EIA/RIA 8-well stripes (Corning Incorporated, Corning, New York, USA). A polyclonal antibody produced in chicken against each of the analytes was used as a secondary antibody. O-phenylenediamine dihydrochloride (OPD) (Sigma, Steinheim, Germany) was used to visualize the peroxidase label. Color formation was measured on 450 nm (Anthos 2001 microplate reader), and calculations were done using a Windows-based control and evaluation software for Rosys Anthos microplate readers (Anthos labtec instruments, Wals, Austria).

 The monoclonal antibody against MMP-2-TIMP-2 recognizes the solube lMMP-2-TIMP-2 complex. The monoclonal antibody against MMP-9 (code Ge-213) recognizes both free MMP-9 and that bound to its inhibitor TIMP-1. The monoclonal antibody against TIMP-1 (code DB-102D1) recognizes both free TIMP-1 and that complexed with MMP-9. It does not cross-react with TIMP-2. The monoclonal antibody against TIMP-2 (code T2-101) recognizes both free TIMP-2 and that complexed with MMP-2.

Each sample was run in duplicate in order to minimize intraassay variation. The absorbance values for standard samples and the standard curves constructed for each assay were compared and used to minimize interassay variation.

### 2.2. Statistical Analysis

 Statistical analysis was performed with SPSS (v. 11.5) for Windows (SPSS Inc, Chicago, Ill. USA) software package. Wilcox test was used to investigate the significance of the differences between medians. A *P*-value <  .05 was considered statistically significant.

## 3. Results

The mean levels of serum TIMP-2 and MMP-2-TIMP-2 complex were higher in the healthy controls compared to those with a malignant tumor (Table 1). For the MMP-9 and TIMP-1, no significant differences were found between patients' and controls' concentrations. 

Serum TIMP-2 values decreased significantly from healthy controls (mean 323 *μ*g/l, range 305–342 *μ*g/l) to malignant (mean 136 *μ*g/l, range 120–151 *μ*g/l) squamous cervical carcinoma patients (*P* < .000). 

Serum proMMP2-TIMP2 complex values decreased from control patients (mean 625 *μ*g/l, range 588–662 *μ*g/l) to malignant (mean 514 *μ*g/l, range 453–575 *μ*g/l) to squamous cervical carcinoma patients (*P* < .006).

## 4. Discussion

This paper shows for the first time that the levels of circulating TIMP-2 and the MMP-2-TIMP-2 complex are lower in squamous cervical cancer patients than in healthy women. Previously Ylisirniö et al. [8] found that the TIMP-2 and the MMP-2/TIMP-2 complex levels were lower in lung cancer than in the sera of the control subjects. Also in colorectal cancer, the serum levels of the MMP-2-TIMP-2 complex are found to be lower than in healthy controls [9]. Paula et al.[10] found that breast cancer patients had significantly lower TIMP-2 levels than did healthy controls.

We found in this study that squamous cervical cancer patients had significantly lower TIMP-2 levels compared to healthy controls. In squamous cervical cancer, no evidence currently exists showing that TIMP-2 could be prognostic when measured in peripheral blood. However, the presence of the disease might alter the balance of the proteolysis. Low TIMP-2 levels in the blood of squamous cervical cancer patients could indicate more activated MMP-2 and therefore higher usage of TIMP-2, or alternatively, the lower levels could be due to lower production of TIMP-2, leading to less inhibition of MMP-2 activity.

For the proMMP2-TIMP2 complex, lower concentrations are found in squamous cervical cancer patients. It has been shown that during aggregation, platelets release MMP-2 in its latent form [11] as well as other components of the proMMP2/MT1-MMP/TIMP-2 system [12].

Patients and controls did not have significant differences in their serum MMP-9 and TIMP-1 levels, although the ranges for MMP-9 and TIMP-1 concentrations were wider in cancer patients compared with healthy controls. This could indicate the presence of a disturbance, such as cancer, that could affect the levels of the analytes.

In conclusion, decreased circulating levels of TIMP-2 and MMP2-TIMP2 complex are found in the serum samples of squamous cervical carcinoma patients. Future studies will determine the prognostic value of TIMP-2 and MMP2-TIMP2 in squamous cervical carcinoma.

## Figures and Tables

**Table 1:** Serum levels of MMP-2-TIMP-2 complex, MMP-9, TIMP-1, and TIMP-2 in patients with squamous cervical cancer and in healthy controls.

Serum marker	Cervical cancer mean (range)	Healthy controls mean (range)	*P*-value^1^
MMP-2-TIMP-2 (*μ*g/l)	514 (453–575)	625 (588–662)	.006
MMP-9 (*μ*g/l)	74 (40–108)	73 (59–87)	.659
TIMP-1 (*μ*g/l)	466 (422–511)	414 (383–443)	.067
TIMP-2 (*μ*g/l)	136 (120–151)	323 (305–342)	.000

The results are expressed as mean (range).^1^ Wilcox
